# High Correlation Between Li^+^ Solvation Energy and Li^+^ Ionic Conductivity in Lithium Metal Battery Electrolytes

**DOI:** 10.3390/ijms252413268

**Published:** 2024-12-10

**Authors:** Jihoon Choi, Young-Kyu Han

**Affiliations:** Department of Energy and Materials Engineering and Advanced Energy and Electronic Materials Research Center, Dongguk University-Seoul, Seoul 04620, Republic of Korea; chchc2059@gmail.com

**Keywords:** lithium metal battery, fluorinated solvent, solvent–ion interaction, ionic conductivity, first-principles calculation

## Abstract

In lithium metal batteries, accurately estimating the Li^+^ solvation ability of solvents is essential for effectively modulating the Li^+^ solvation sheath to form a stable interphase and achieve high ionic conductivity. However, previous studies have shown that the theoretically calculated Li^+^ binding energy, commonly used to evaluate solvation ability, exhibits only a moderate correlation with experimentally measured ionic conductivity (R^2^ = 0.68). In this study, to determine the effective theoretical descriptor for evaluating the solvation ability, Li^+^ solvation energy was adopted instead of Li^+^ binding energy, and its correlation with ionic conductivity was compared. Using a sophisticated calculation model that considers the Li^+^ counter anion and solvent, it was demonstrated that the tendency between the calculated Li^+^ solvation energies and experimentally measured ionic conductivities is highly consistent (R^2^ = 0.97). Therefore, Li^+^ solvation energy is suggested as the theoretical descriptor for evaluating solvation ability. All these findings encourage the development of effective molecular design of solvents for lithium metal batteries.

## 1. Introduction

Lithium metal is a highly promising anode material for next-generation batteries due to its high theoretical specific capacity (3860 mAh g^−1^) and low negative electrochemical potential (−3.04 V vs. the standard hydrogen electrode) [1]. Despite these outstanding advantages, lithium metal batteries suffer from poor cycle stability and low Coulombic efficiency owing to an irreversible Li plating/stripping process [2]. In particular, the solid electrolyte interphase (SEI) that forms on the surface of the lithium anode in lithium metal batteries is prone to cracking during the charge and discharge cycles, leading to the formation of Li dendrites [3]. These dendrites are so-called “dead Li,” as they represent an irreversible loss of available lithium, which continuously degrades the cycling performance [4]. Dendrite formation is a critical issue, not only in Li metal batteries but also in sodium-ion batteries, and the Na dendrites formed during the cycling process are the main factors inhibiting performance improvement [5]. Addressing these issues in lithium metal batteries is a challenging task, as the solution requires the formation of a stable SEI on the Li anode surface while inhibiting the generation of Li dendrites [6].

The problems of lithium metal anodes have been addressed by various electrolyte engineering approaches that involve the formation of an artificial but stable SEI [7]. One widely reported approach has been to introduce fluorine atoms into organic solvents to enhance battery performance [8]. Fluorinated organic solvents are known for their high oxidative stability and relatively low solvation ability, which encourages the bis(fluorosulfonyl)imide (FSI) anion to participate in the Li^+^ solvation sheath and promotes the formation of a stable anion-derived SEI [9]. However, a drawback of fluorinated organic solvents is their significantly lower ionic conductivity than commercial carbonate electrolytes, which allows the battery to operate only at low charging rates [10]. Furthermore, environmental concerns regarding solvents with excessive amounts of introduced fluorine atoms may restrict their commercial application in the battery industry [11]. Therefore, a crucial issue in electrolyte engineering is achieving a balance between oxidative stability and ionic conductivity by introducing optimal fluorinated groups and regulating the solvation ability.

Many researchers have addressed these issues by introducing only one or two fluorine atoms into the solvent to balance oxidative stability and ionic conductivity and promote high cycling performance [12,13,14,15,16,17]. Introducing a –CF_3_ group into the solvent produces very high oxidative stability but leads to poor ionic conductivity due to weak solvation. By contrast, introducing a locally polar –CH_2_F or –CHF_2_ group into the solvent enables relatively strong Li^+^–solvent interaction, resulting in higher ionic conductivity compared to solvents with a –CF_3_ group.

If the interaction energy between the solvent and the cation salt (Li^+^) is high, the strong interactions between the solvent and Li^+^ reduce the pairing between the lithium ion and the anion salt. Consequently, the number of separated mobile Li^+^ charge carriers increases, leading to a higher ionic conductivity [14,15,18]. However, other factors can also influence ionic conductivity, such as the interactions between the solvent and the anion salt, the size of the solvated Li^+^ cluster ions, and the solvent coordination number for Li^+^ [19,20,21].

In the literature, the Li^+^ solvation ability has been analyzed using ionic conductivity measured experimentally or by calculating the Li^+^ binding energy of the solvent using density functional theory (DFT) [12,13,14,15,16,17]. A higher ionic conductivity or Li^+^ binding energy is interpreted as indicating a higher solvation ability. Zhang et al. synthesized a bis(2-fluoroethyl) ether (BFE) by introducing a –CH_2_F group into a conventional diethyl ether (DE) solvent. The BFE-based electrolyte formed a stable SEI on the Li anode surface in lithium metal batteries and had high ionic conductivity [12]. They also used the Li^+^ binding energy calculated using DFT calculations and the ionic conductivity value measured experimentally to explain the Li^+^ solvation ability of the solvent molecules effectively. This low degree of fluorination has been applied to various solvent systems other than ether-based solvents. Wang et al. used 2,2-difluoroethyl acetate (DFEA) as a solvent to introduce a –CHF_2_ group into ethyl acetate. They demonstrated that DFEA induced higher ionic conductivity and better cycling performance compared to 2,2,2-trifluoroethyl acetate (TFEA), which introduces a –CF_3_ group into ethyl acetate [13]. Calculation of the Li^+^ binding energy using DFT showed that the Li^+^ binding energy was higher for DFEA than for TFEA, and the Li^+^ binding energy and ionic conductivity values were used to effectively explain the Li^+^ solvation ability of the solvent molecules.

The present study addressed inconsistencies reported in previous papers regarding the correlation between the calculated Li^+^ binding energies and experimentally measured ionic conductivities. For example, Ruan et al. synthesized 1,2-bis(2-fluoroethoxy) ethane (FDEE) by introducing a –CH_2_F group into a conventional 1,2-diethoxy ethane (DEE) solvent. The resulting FDEE-based electrolytes had a stable anion-derived SEI and a high cycling performance [14]. The stronger solvation ability of FDEE than 1,2-bis(2-chlororthoxy) ethane (ClDEE) was explained by the higher ionic conductivity of FDEE than ClDEE. Their DFT calculations showed that the Li^+^ binding energy was higher for FDEE than for DEE, indicating a stronger solvation ability for FDEE containing the –CH_2_F group. However, the ionic conductivity was higher for DEE than for FDEE, showing a qualitative inconsistency between Li^+^ binding energy and ionic conductivity. Similarly, Yu et al. introduced fluorine atoms into a conventional DEE solvent and then introduced a –CHF_2_ group to induce stronger Li^+^ solvation ability than was achievable with the molecule with the –CF_3_ group. They obtained high cycle performance from the 1,2-bis(2,2-difluoroethoxy) ethane (F4DEE) molecule with the –CHF_2_ group [15]. DFT calculations demonstrated that the Li^+^ binding energy was higher for the molecules with the –CHF_2_ group than for the molecules with the –CF_3_ group, indicating stronger solvation ability for the molecules containing a –CHF_2_ group. However, a comparison of DEE and F4DEE in the same paper revealed a higher Li^+^ binding energy for F4DEE but a higher ionic conductivity for DEE, showing a qualitative inconsistency between the two descriptors. Mo et al. synthesized ethyl monofluoroacetate (EFA) by introducing a –CH_2_F group into the conventional ethyl acetate (EA) solvent and were able to induce a stable anion-derived SEI and relatively higher cycle performance compared to ethyl trifluoroacetate (ETFA) with –CF_3_ group [16]. Their DFT calculations revealed a higher Li^+^ binding energy for EFA than for ETFA, and the ionic conductivity was higher for EFA, thereby explaining the stronger solvation ability for EFA. However, a comparison of EA and EFA revealed a higher Li^+^ binding energy for EFA but a higher ionic conductivity for EA, indicating a different tendency between the two descriptors. Yu et al. synthesized monofluoroethyl methyl carbonate (F1EMC) by introducing a –CH_2_F group into the conventional ethyl methyl carbonate (EMC) solvent. They found that the resulting F1EMC-based electrolytes induced a stable anion-derived SEI and showed a higher cycling performance compared to trifluoroethyl methyl carbonate (F3EMC) with a –CF_3_ group [17]. Their DFT calculations showed that the Li^+^ binding energy was higher for F1EMC than for F3EMC, and the ionic conductivity was higher for F1EMC, indicating a stronger solvation ability for F1EMC. However, a comparison of EMC and F1EMC in the same paper revealed that the Li^+^ binding energy was higher for F1EMC, while the ionic conductivity was higher for EMC, again showing a qualitative inconsistency between the two descriptors.

Li^+^ binding energy and ionic conductivity have been used to evaluate and compare the solvation ability of solvents. However, many of the reported results indicate different tendencies of Li^+^ binding energy and ionic conductivity, leaving in question whether a correlation exists between Li^+^ binding energy and ionic conductivity and, if so, how strong the correlation is. This uncertainty raises the requirement for an effective theoretical descriptor to evaluate the solvation ability.

In the present work, to determine the effective theoretical descriptor for evaluating the solvation ability, Li^+^ solvation energy was adopted instead of Li^+^ binding energy, and the level of correlation between the Li^+^ solvation energy of solvents and the experimentally measured ionic conductivity was clarified by calculating the Li^+^ solvation energies of a solvent using a model that considered the Li^+^ counter anion and the solvent in the solvation sheath. In the previous literature, the calculated Li^+^ binding energies for the Li^+^:solvent = 1:1 complex in the gas phase often exhibited divergent tendencies from the experimentally measured ionic conductivity. However, our computational model, which considers the Li^+^ counter anion and the solvent, demonstrates a complete qualitative consistency between Li^+^ solvation energy and ionic conductivity. The correlation coefficient between ionic conductivity and Li^+^ solvation energy calculated from our model is significantly higher, with an R^2^ = 0.97, compared to the lower R^2^ = 0.68 reported in the previous studies. A fairly high correlation between Li^+^ solvation energy and ionic conductivity was demonstrated, indicating that Li^+^ solvation energy can be used as an effective descriptor for evaluating the solvation ability of solvents. In particular, when evaluating solvation ability, it is suggested that countless candidate molecules can be screened through calculations using the Li^+^ solvation energy.

## 2. Results and Discussion

**Figure 1:**
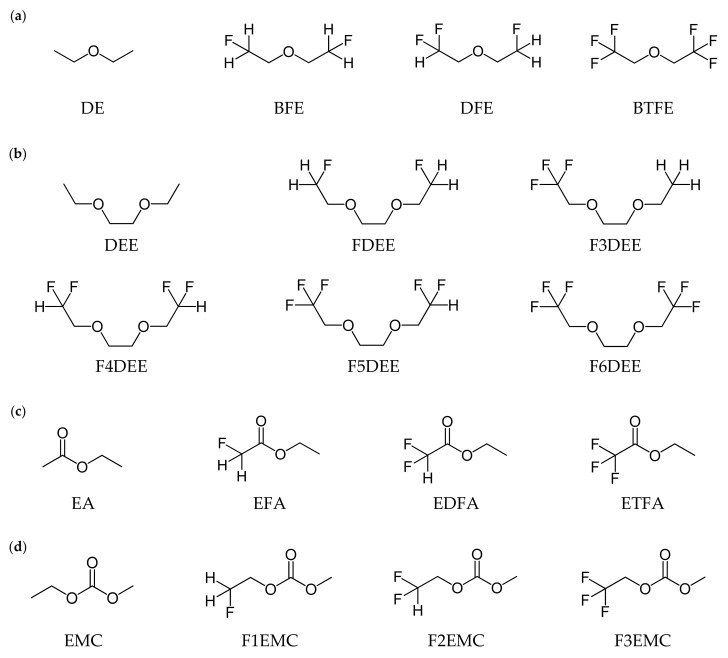
2D structures of the pristine and fluorinated solvent molecules. The molecules are divided into ether-based groups: (**a**) DE, BFE, 1,1-difluoroethyl-2-fluoroethyl ether (DFE), and bis(2,2,2-trifluoroethyl) ether (BTFE); (**b**) DEE, FDEE, ethoxy-2,2,2-trifluoroethoxy ethane (F3DEE), F4DEE, 1-(2,2-difluoroethoxy)-2-(2,2,2-trifluoroethoxy) ethane (F5DEE), and 1,2-bis(2,2,2-trifluoroethoxy) ethane (F6DEE), ethyl acetate-based group; (**c**) EA, EFA, ethyl difluoroacetate (EDFA), and ETFA, and carbonate-based group; (**d**) EMC, F1EMC, difluoroethyl methyl carbonate (F2EMC), and F3EMC.

**Figure 2:**
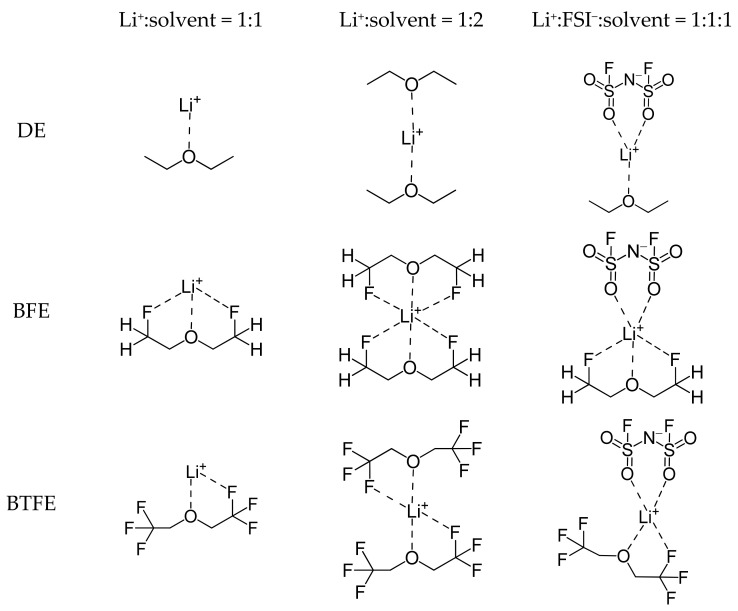
2D structures of the Li^+^ solvation sheath for the Li^+^:solvent = 1:1, Li^+^:solvent = 1:2, and Li^+^:FSI^−^:solvent = 1:1:1 complexes of DE, BFE, and BTFE.

DFT-based calculations were performed on 18 molecules (Figure 1) that had been evaluated in previous studies to analyze the correlation between Li^+^ solvation energy and ionic conductivity. The optimized structures of the considered molecules are shown in Figure S1. To determine how the Li^+^ solvation energies vary depending on the composition of the Li^+^ solvation sheath, the Li^+^ solvation energy was calculated for three types of complexes (Li^+^:solvent = 1:1, Li^+^:solvent = 1:2, and Li^+^:FSI^−^:solvent = 1:1:1 complexes) of the Li^+^ solvation sheath, as shown in Figure 2. The optimized structures of the Li^+^ solvation sheath are shown in Figures S2–S5. A linear regression analysis of the Li^+^ solvation energy and ionic conductivity was also performed to further evaluate the correlation coefficient.

### 2.1. Li^+^ Solvation Energies According to the Solvation Sheath Compositions

**Figure 3:**
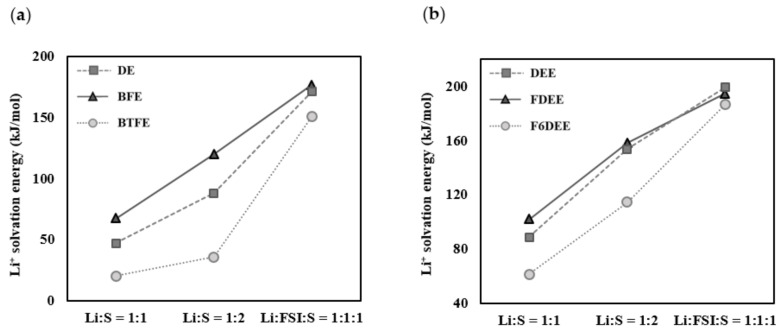
Li^+^ solvation energies of (**a**) DE, BFE, and BTFE and (**b**) DEE, FDEE, and F6DEE for the Li^+^:solvent = 1:1, Li^+^:solvent = 1:2, and Li^+^:FSI^−^:solvent = 1:1:1 complexes.

The molecules shown in Figure 1 were placed into four groups—group Ⅰ (DE, BFE, and BTFE), group Ⅱ (DEE, FDEE, and F6DEE), group Ⅲ (EA, EFA, and ETFA), and group Ⅳ (EMC, F1EMC, and F3EMC)—to compare Li^+^ solvation energies in the Li^+^:solvent = 1:1, Li^+^:solvent = 1:2, and Li^+^:FSI^−^:solvent = 1:1:1 complexes (Figure 2). Each group includes a pristine molecule, a molecule with a –CH_2_F group, and a molecule with a –CF_3_ group. As presented in Figure 3a, the Li^+^ solvation energy tendencies appear to be the same for all compositions, and a comparison of DE and BFE indicates that the Li^+^ solvation energy and ionic conductivity show an order of BFE > DE and have identical tendency (Table 1). However, as shown in Figure 3b, the Li^+^ solvation energy tendencies appear different depending on the composition. As shown in Table 1, a comparison of DEE and FDEE indicates that the Li^+^ solvation energies for the Li^+^:FSI^−^:solvent = 1:1:1 complex show the order of DEE > FDEE and have the identical tendency for ionic conductivities, but the Li^+^ solvation energies for the Li^+^:solvent = 1:1 and Li^+^:solvent = 1:2 complexes show the order of DEE < FDEE and have a different tendency from the ionic conductivities. This discrepancy is because the number of oxygen and fluorine atoms coordinating with Li^+^ varies depending on the number and type of molecules coordinating around Li^+^ in the solvation sheath, thereby influencing the interaction between Li^+^ and the surrounding solvent and anion salt.

**Figure 4:**
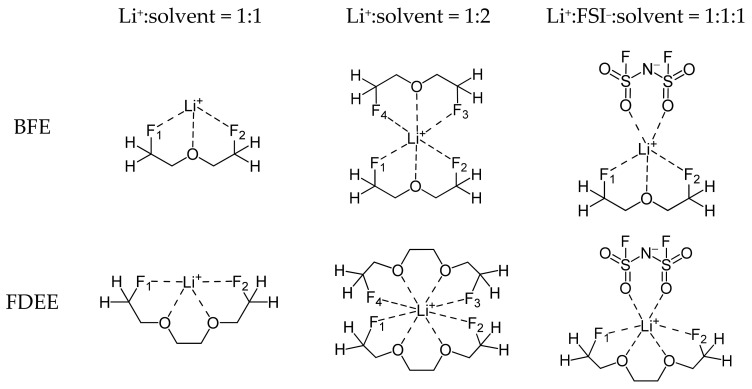
2D structures of the ether-based solvents having a –CH_2_F group for the Li^+^:solvent = 1:1, Li^+^:solvent = 1:2, and Li^+^:FSI^−^:solvent = 1:1:1 complexes.

How the influence of oxygen and fluorine in the solvent changes depending on the number and type of molecules coordinating around Li^+^ in the solvation sheath was analyzed by comparing the Li^+^–F bond lengths for the Li^+^:solvent = 1:1, Li^+^:solvent = 1:2, and Li^+^:FSI^−^:solvent = 1:1:1 complexes in ether-based molecules with a –CH_2_F group. The 2D structures specifying the positions of F_1_–F_4_ in the solvent with the –CH_2_F group are shown in Figure 4. As shown in Table 2, which specifies the bond lengths of Li^+^ and F_1_–F_4_, the Li^+^–F bond length is shorter for BFE and FDEE with the –CH_2_F group in the Li^+^:solvent = 1:1 complex than for other complexes. However, in the Li^+^:solvent = 1:2 and Li^+^:FSI^−^:solvent = 1:1:1 complexes, the Li^+^–F bond length appears relatively long. This means that in the Li^+^:solvent = 1:1 complex, the interaction between Li^+^ and fluorine atoms appears relatively strong, while in the Li^+^:solvent = 1:2, Li^+^:FSI^−^:solvent = 1:1:1 complexes, the interaction between Li^+^ and fluorine atoms appears relatively weak. As shown in Figure S6, the Li^+^ solvation energy of the structure where Li^+^ is coordinated to the oxygen of solvent is higher than the structure where Li^+^ is coordinated to fluorine. The NBO charge distribution in Figure S7 shows oxygen atoms have a more negative charge than fluorine atoms in both BFE and FDEE. This explains why the Li^+^ solvation energy is higher for the structure where Li^+^ is coordinated with the oxygen atoms of the solvent. Since the interaction with Li^+^ is stronger for oxygen than for fluorine, when the Li cation is stabilized by interacting with multiple oxygens, such as in the Li^+^:solvent = 1:2, Li^+^:FSI^−^:solvent = 1:1:1 complexes, the interaction between fluorine and Li^+^ is somewhat weakened, which can be interpreted as a lengthening of the Li^+^–F bond length. In particular, when two oxygens in one molecule can contribute to the interaction with Li^+^, as in FDEE, the degree of stabilization of Li^+^ by the oxygen in the solvent becomes substantial, which can be interpreted as a considerable weakening of the influence of fluorine and an increase in the Li^+^–F bond length. The weak interaction between Li^+^ and fluorine atoms in the Li^+^:solvent = 1:2 and Li^+^:FSI^−^:solvent = 1:1:1 complexes was further confirmed through bond energy analysis. The detailed results of this analysis were included in the Supporting Information (Figures S8 and S9, Table S1). In the case of solvents containing fluorine, these characteristics mean that, in the Li^+^:FSI^−^:solvent = 1:1:1 complex, where the Li cation can interact with the oxygen of the solvent and FSI anion, the interaction between the –CH_2_F group and Li^+^ is considerably weakened, and the Li^+^ solvation energy is significantly reduced.

**Figure 5:**
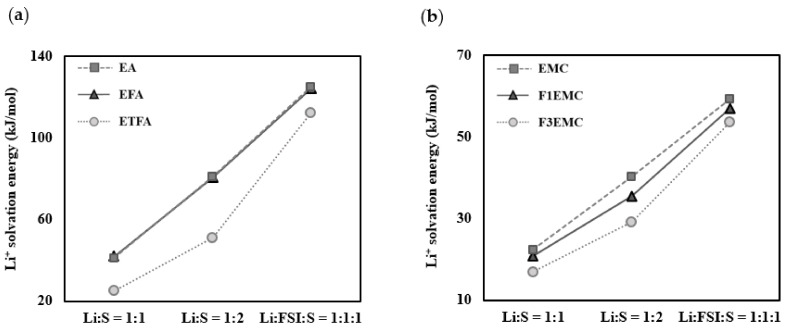
Li^+^ solvation energies of (**a**) EA, EFA, and ETFA and (**b**) EMC, F1EMC, and F3EMC for the Li^+^:solvent = 1:1, Li^+^:solvent = 1:2, and Li^+^:FSI^−^:solvent = 1:1:1 complexes.

Whether the results showing different Li^+^ solvation energy tendencies depending on the number and type of molecules coordinating around Li^+^ in the solvation sheath also appear in solvents other than ether-based solvents was investigated by comparing the Li^+^ solvation energies for the Li^+^:solvent = 1:1, Li^+^:solvent = 1:2, and Li^+^:FSI^−^:solvent = 1:1:1 complexes in ethyl acetate-based and carbonate-based solvents. As shown in Figure 5a, the Li^+^ solvation energy tendencies appear to differ depending on the composition. As shown in Table 3, a comparison of EA and EFA indicates that the Li^+^ solvation energies for the Li^+^:solvent = 1:2, Li^+^:FSI^−^:solvent = 1:1:1 complexes show the order of EA > EFA > ETFA, and have an equal tendency with ionic conductivity. However, in the Li^+^:solvent = 1:1 complex, the Li^+^ solvation energies show the order EFA > EA > ETFA and have a different tendency to that of the ionic conductivity.

As presented in Figure 5b, the Li^+^ solvation energies show the order of EMC > F1EMC > F3EMC in all compositions and appear to have an identical tendency to that of the ionic conductivity. Unlike the calculation results of previous studies, the Li^+^ solvation energy and ionic conductivity show an identical tendency even in the Li^+^:solvent = 1:1 complex. This consistency is attributed to using a more sophisticated calculation model in our research, which considers the bulk solvent effect. In ethyl acetate and carbonate-based solvents, the oxygen in the C=O bond also contributes significantly to stabilizing Li^+^, just like the oxygen in the C–O–C bond does in the ether-based solvent. Thus, when more oxygen contributes to the interaction with Li^+^, the influence of fluorine would be weakened. The calculation results for groups Ⅰ–IV show that the Li^+^ solvation energies calculated for the Li^+^:FSI^−^:solvent = 1:1:1 complex show completely consistent results with the experimental ionic conductivities. 

### 2.2. Linear Regression Analysis of the Correlation Between Li^+^ Solvation Energies and Ionic Conductivities

The correlation between Li^+^ solvation energy and ionic conductivity in the various Li^+^ solvation sheath compositions was numerically confirmed through a linear regression analysis between Li^+^ solvation energy and ionic conductivity. The molecules shown in Figure 1, whose ionic conductivity was measured and reported under the same experimental conditions, were grouped into four groups: group A (DE, BFE, and DFE); B (DEE, F3DEE, F4DEE, F5DEE, and F6DEE); C (EA, EFA, EDFA, and ETFA); and D (EMC, F1EMC, F2EMC, and F3EMC). Since ionic conductivity is a value that changes depending on experimental conditions, comparing the ionic conductivity and calculated values is meaningless when the experimental conditions are different. The calculated Li^+^ solvation energy and experimental ionic conductivity for each group are specified in Table 4, and the correlation coefficient (R^2^) values obtained by performing the linear regression analysis between the Li^+^ solvation energy and ionic conductivity are listed in Table 5. The linear regression plot between the Li^+^ solvation energy and ionic conductivity for the Li^+^:solvent = 1:1, Li^+^:solvent = 1:2, and Li^+^:FSI^−^:solvent = 1:1:1 complexes are presented in Figures S10–S13. The correlation coefficient values in Table 5 increase in the following order: Li^+^:solvent = 1:1 < Li^+^:solvent = 1:2 < Li^+^:FSI^−^:solvent = 1:1:1 for groups A–D.

The correlation coefficient between the Li^+^ solvation energy for the Li^+^:solvent = 1:1 complex calculated from previous research and the experimental value for ionic conductivity shows the following values: group A (0.99), group B (0.28), group C (0.76), and group D (0.70). Group A showed a very high correlation coefficient, while the other groups showed relatively low values. The correlation was especially low in the case of group B. However, our calculation results showed fairly high correlation coefficient values for all four groups, especially for groups A and B, where the correlation coefficient values showed very high values of 0.998 and 0.996 for the Li^+^:FSI^−^:solvent = 1:1:1 complex. In addition, because the bulk solvent effect, which was not considered in previous studies, was considered in the present work, our calculation results show a higher correlation coefficient in the case of groups B, C, and D, even for the Li^+^:solvent = 1:1 complex, which has often shown different tendencies between Li^+^ solvation energy and ionic conductivity. The previous study results showed a low value of R^2^ = 0.68, while the calculation results of this study show high values: Li^+^:solvent = 1:1 (0.91) < Li^+^:solvent = 1:2 (0.96) < Li^+^:FSI^−^:solvent = 1:1:1 (0.97). In particular, presented linear regression analysis shows a very high correlation coefficient, proving that a fairly high correlation exists between Li^+^ solvation energy and ionic conductivity. The most accurate method to calculate the ionic conductivity of Li^+^ in the secondary battery electrolyte would be to perform ab initio molecular dynamics (AIMD) simulations [22,23]. However, these calculations, which explicitly consider all molecules within the electrolyte, are technically challenging and highly time-consuming. It can be said that previous studies calculated the binding energy by considering only the interaction between Li^+^ and the solvent as the main interaction. However, through the results of this study, we learned that it is necessary to describe at least the first solvation sheath explicitly while implicitly considering the bulk solvent effect. We suggest that the Li^+^:FSI^−^:solvent = 1:1:1 complex model is the simplest model capable of providing meaningful results. In other words, the calculated value of the Li^+^ solvation energy is a descriptor that can explain the solvation ability at a similar level to that possible using the experimental value of ionic conductivity.

## 3. Materials and Methods

A computational study based on Kohn–Sham density functional theory was conducted to structurally optimize the Li^+^ solvation sheath and analyze the interaction between the organic solvent and Li cation [24,25]. DFT calculations were performed using the B3LYP functional [26,27] and the 6-311++G(d,p) basis set in the Gaussian16 software revision C. 01 package [28]. The B3LYP function is widely used to accurately describe the structure of the complex and electrostatic interactions in lithium metal batteries [29,30]. The influence of the bulk solvent on the molecular structure and electrical properties was considered using the polarizable continuum model (PCM), one of the self-consistent reactive field methodologies [31,32]. The dielectric constants of the various molecules used in the calculations were considered by employing the dielectric constant ε = 4.24 of diethyl ether for ether-based molecules [33,34] and the dielectric constant ε = 20.5 of acetone for carbonate-based molecules [35,36]. For ethyl acetate-based molecules, the dielectric constant ε = 6.25 of acetic acid, which has a similar structure and dielectric constant to ethyl acetate (6.02), was used [37]. Natural bond orbital (NBO) analysis was performed using NBO 7.0 [38] at the B3LYP/6-311++G(d,p) level, considering the bulk solvent effect to determine the NBO charge distribution on the individual atoms. The Li^+^ solvation energy (E_b_) between Li^+^ and a specific solvent or anion salt in the complexes was calculated using Equations (1)–(3), while the E_b_ for the Li^+^:solvent = 1:1, Li^+^:solvent = 1:2, and Li^+^:FSI^−^:solvent = 1:1:1 complexes were obtained using Equations (1)–(3), respectively.

E_b_ = −[E(Li^+^–solvent complex) − E(Li^+^) − E(solvent)] (1)

E_b_ = −[E(Li^+^(solvent)_2_ complex) − E(Li^+^) − 2 × E(solvent)] (2)

E_b_ = −[E(FSI^−^–Li^+^–solvent complex) − E(Li^+^) − E(FSI^−^) − E(solvent)] (3)

## 4. Conclusions

The Li^+^ solvation energies for various compositions of the solvation sheath were calculated to analyze the correlation between the Li^+^ solvation energy and the experimentally measured ionic conductivity. While the Li^+^ binding energy in the Li^+^:solvent = 1:1 complex reported in previous studies exhibited a low correlation with the experimental ionic conductivity, the Li^+^ solvation energy in the Li^+^:FSI^−^:solvent = 1:1:1 complex calculated in this study accurately considers the interaction between organic solvents and Li^+^, demonstrating a very high correlation with ionic conductivity. The high correlation between the Li^+^ solvation energy and ionic conductivity confirms that Li^+^ solvation energy is an effective descriptor for interpreting the solvation ability of a solvent. When studying solvents with controlled solvation ability for advanced electrolyte design for Li metal batteries, our research findings contribute to effectively predicting the solvation ability of solvents in the molecular design process. Advanced solvents, developed through an appropriate molecular design process, can facilitate the formation of a stable and uniform SEI, thereby extending the lifespan of lithium metal batteries. Furthermore, it is suggested that countless candidate substances can be screened using calculations alone and the Li^+^ solvation energy. The findings of this study suggest that Li^+^ solvation energy can be utilized as a main feature in developing a machine-learning model based on quantum chemistry to provide a relatively accurate prediction of ionic conductivity and solvation ability.

## Figures and Tables

**Table 1:** The experimental ionic conductivities (ms cm^−1^) and calculated Li^+^ solvation energies (kJ/mol) of ether-based solvents for the Li^+^:solvent = 1:1, Li^+^:solvent = 1:2, and Li^+^:FSI^−^:solvent = 1:1:1 complexes.

Molecule	Li^+^ Solvation Energy			Ionic Conductivity
	Li^+^:Solvent = 1:1	Li^+^:Solvent = 1:2	Li^+^:FSI^−^:Solvent = 1:1:1	Experiment (Reference)
DE	47.3	88.1	171.4	1.0 ^a^
BFE	67.4	120.2	176.6	7.4 ^a^
BTFE	20.4	35.9	150.9	–
DEE	88.8	153.9	199.3	7.2 ^b^
FDEE	102.0	158.7	194.7	3.2 ^b^
F6DEE	61.6	114.7	186.8	–

^a^ Measured in 1 M lithium bis(fluorosulfonyl)imide (LiFSI)/solvent at 30 °C [12]. ^b^ Measured in LiFSI:solvent:1,1,2,2-tetrafluoroethyl-2,2,3,3-tetrafluoropropyl ether (TTE) = 1:1.6:3 (molar ratio) at 25 °C [14].

**Table 2:** The calculated Li^+^−F bond lengths (Å) for the Li^+^:solvent = 1:1, Li^+^:solvent = 1:2, and Li^+^:FSI^−^:solvent = 1:1:1 complexes.

Molecule	Li^+^−F Bond Length		
	Li^+^:Solvent = 1:1	Li^+^:Solvent = 1:2	Li^+^:FSI^−^:Solvent = 1:1:1
BFE			
Li^+^–F_1_	2.08	2.12	2.11
Li^+^–F_2_	2.15	2.17	2.13
Li^+^–F_3_		2.11	
Li^+^–F_4_		2.15	
FDEE			
Li^+^–F_1_	2.08	2.94	2.78
Li^+^–F_2_	2.08	3.16	2.70
Li^+^–F_3_		3.12	
Li^+^–F_4_		3.14	

**Table 3:** The experimental ionic conductivities (ms cm^−1^) and calculated Li^+^ solvation energies (kJ/mol) of ethyl acetate and carbonate-based solvents for the Li^+^:solvent = 1:1, Li^+^:solvent = 1:2, and Li^+^:FSI^−^:solvent = 1:1:1 complexes.

Molecule	Li^+^ Solvation Energy			Ionic Conductivity
	Li^+^:Solvent = 1:1	Li^+^:Solvent = 1:2	Li^+^:FSI^−^:Solvent = 1:1:1	Experiment (Reference)
EA	41.2	81.0	125.1	11.2 ^a^
EFA	41.8	80.6	124.0	8.9 ^a^
ETFA	25.0	51.0	112.3	4.2 ^a^
EMC	22.5	40.4	59.5	0.54 ^b^
F1EMC	20.7	35.5	57.1	0.52 ^b^
F3EMC	17.1	29.3	53.8	0.42 ^b^

^a^ Measured in 1 M LiFSI/solvent:fluoroethylene carbonate (FEC)=9:1 (volume ratio) at 25 °C [16] ^b^ Measured in 1 M lithium hexafluorophosphate (LiPF_6_)/solvent:FEC = 7:3 (volume ratio) [17].

**Table 4:** The experimental ionic conductivity (ms cm^−1^) and calculated Li^+^ solvation energy (kJ/mol) of group A−D for the Li^+^:solvent = 1:1, Li^+^:solvent = 1:2, and Li^+^:FSI^−^:solvent = 1:1:1 complexes.

Molecule	Li^+^ Solvation Energy			Ionic Conductivity
	Li^+^:Solvent = 1:1	Li^+^:Solvent = 1:2	Li^+^:FSI^−^:Solvent = 1:1:1	Experiment (Reference)
DE	47.3	88.1	171.4	1.0 ^a^
BFE	67.4	120.2	176.6	7.4 ^a^
DFE	56.9	98.5	172.4	2.6 ^a^
DEE	88.8	153.9	199.3	11.0 ^b^
F3DEE	73.2	130.5	191.1	6.2 ^b^
F4DEE	76.5	119.4	187.2	4.8 ^b^
F5DEE	67.6	123.4	187.8	5.0 ^b^
F6DEE	61.6	114.7	186.8	4.5 ^b^
EA	41.2	81.0	125.1	11.2 ^c^
EFA	41.8	80.6	124.0	8.9 ^c^
EDFA	32.6	65.6	117.7	7.9 ^c^
ETFA	25.0	51.0	112.3	4.2 ^c^
EMC	22.5	40.4	59.5	0.54 ^d^
F1EMC	20.7	35.5	57.1	0.52 ^d^
F2EMC	18.0	33.1	55.9	0.50 ^d^
F3EMC	17.1	29.3	53.8	0.42 ^d^

^a^ Measured in 1 M lithium bis(fluorosulfonyl)imide (LiFSI)/solvent at 30 °C [12]; ^b^ Measured in 1.2 M LiFSI/solvent [15]; ^c^ Measured in 1 M LiFSI/solvent:fluoroethylene carbonate (FEC) = 9:1 (volume ratio) at 25 °C [16]; ^d^ Measured in 1 M lithium hexafluorophosphate (LiPF_6_)/solvent:FEC = 7:3 (volume ratio) [17].

**Table 5:** The calculated correlation coefficients (R^2^) for groups A−D and the average correlation coefficients for the Li^+^:solvent = 1:1, Li^+^:solvent = 1:2, and Li^+^:FSI^–^:solvent = 1:1:1 complexes.

Group	Correlation Coefficient (R^2^)			
	Li^+^:Solvent = 1:1	Li^+^:Solvent = 1:2	Li^+^:FSI^−^:Solvent = 1:1:1	Li^+^:Solvent = 1:1 (Reference)
A	0.968	0.997	0.998	0.986 ^a^
B	0.871	0.986	0.996	0.278 ^b^
C	0.921	0.940	0.950	0.763 ^c^
D	0.863	0.918	0.931	0.698 ^d^
	**Average correlation coefficient**			
A−D	0.906	0.960	0.969	0.681

^a^ [12]; ^b^ [15]; ^c^ [16]; ^d^ [17].

## Data Availability

The data used to support the findings of this study are available from the corresponding author upon request.

## Supplementary Materials

The following supporting information can be downloaded at: https://www.mdpi.com/article/10.3390/ijms252413268/s1.
