# Supplementary material for: High Correlation Between Li+ Solvation Energy and Li+ Ionic Conductivity in Lithium Metal Battery Electrolytes
Source: Int J Mol Sci. 2024 Dec 10;25(24):13268. doi: 10.3390/ijms252413268 (PMC11678500; doi:10.3390/ijms252413268)
Supplement: Supplementary file 1 [file ijms-25-13268-s001.zip › ijms-3333892-supplementary.pdf]

Supplementary data for

High correlation between Li^+ solvation energy and Li^+ ionic conductivity in lithium metal battery electrolytes

*Jihoon Choi and Young-Kyu Han**

Department of Energy and Materials Engineering and Advanced Energy and Electronic Materials Research Center, Dongguk University-Seoul, Seoul 04620, Republic of Korea

* Corresponding Author

E-mail: ykenergy@dongguk.edu

Figure S1. Optimized structures of (a) DE, BFE, DFE, and BTFE, (b) DEE, FDEE, F3DEE, F4DEE, F5DEE, and F6DEE, (c) EA, EFA, EDFA, and ETFA, and (d) EMC, F1EMC, F2EMC, and F3EMC.

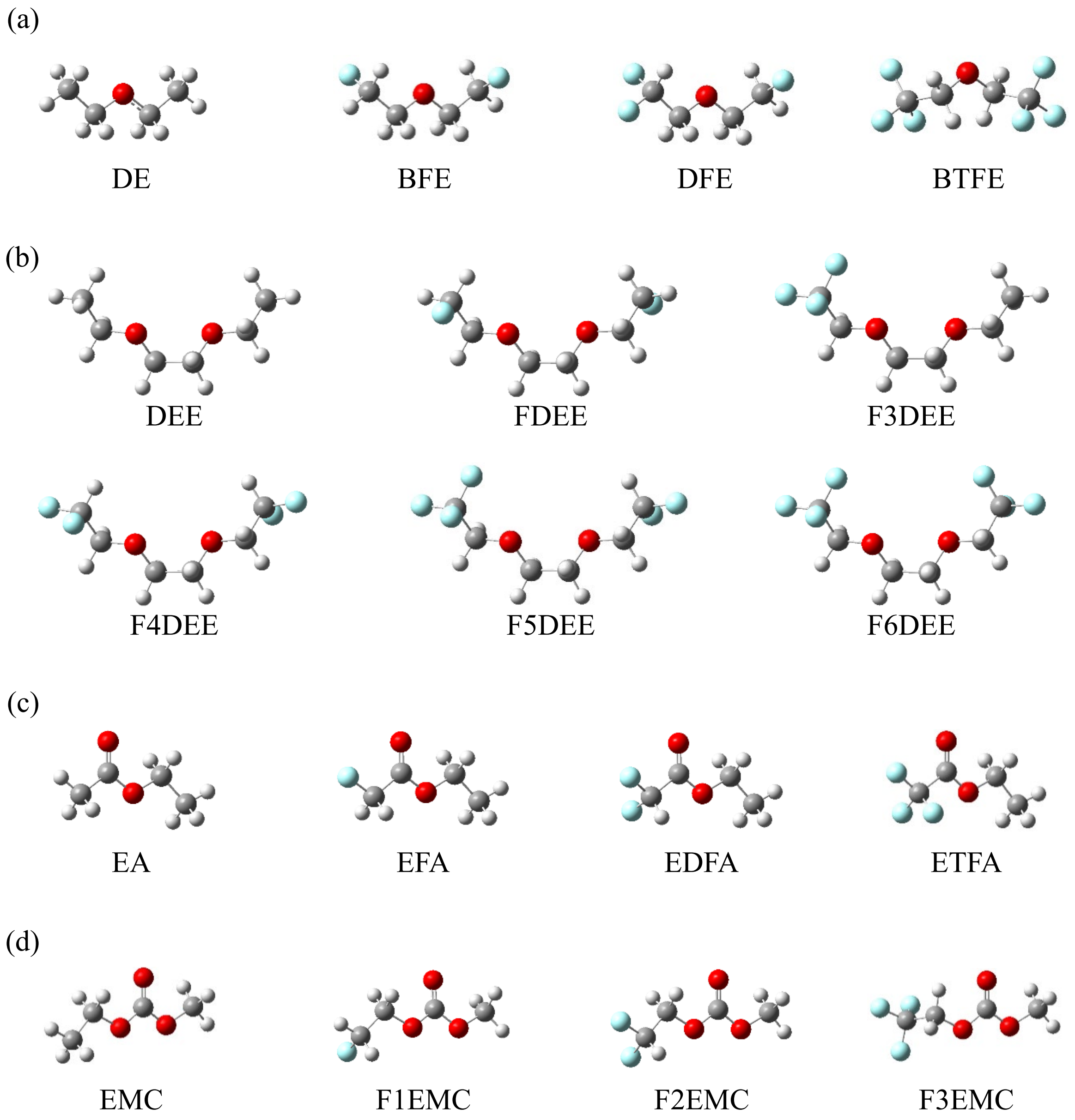

Figure S2. Optimized structures of the Li^+ solvation sheath for the Li^+:solvent = 1:1, Li^+:solvent = 1:2, and Li^+:FSI^-:solvent = 1:1:1 complexes at DE, BFE, DFE, and BTFE.

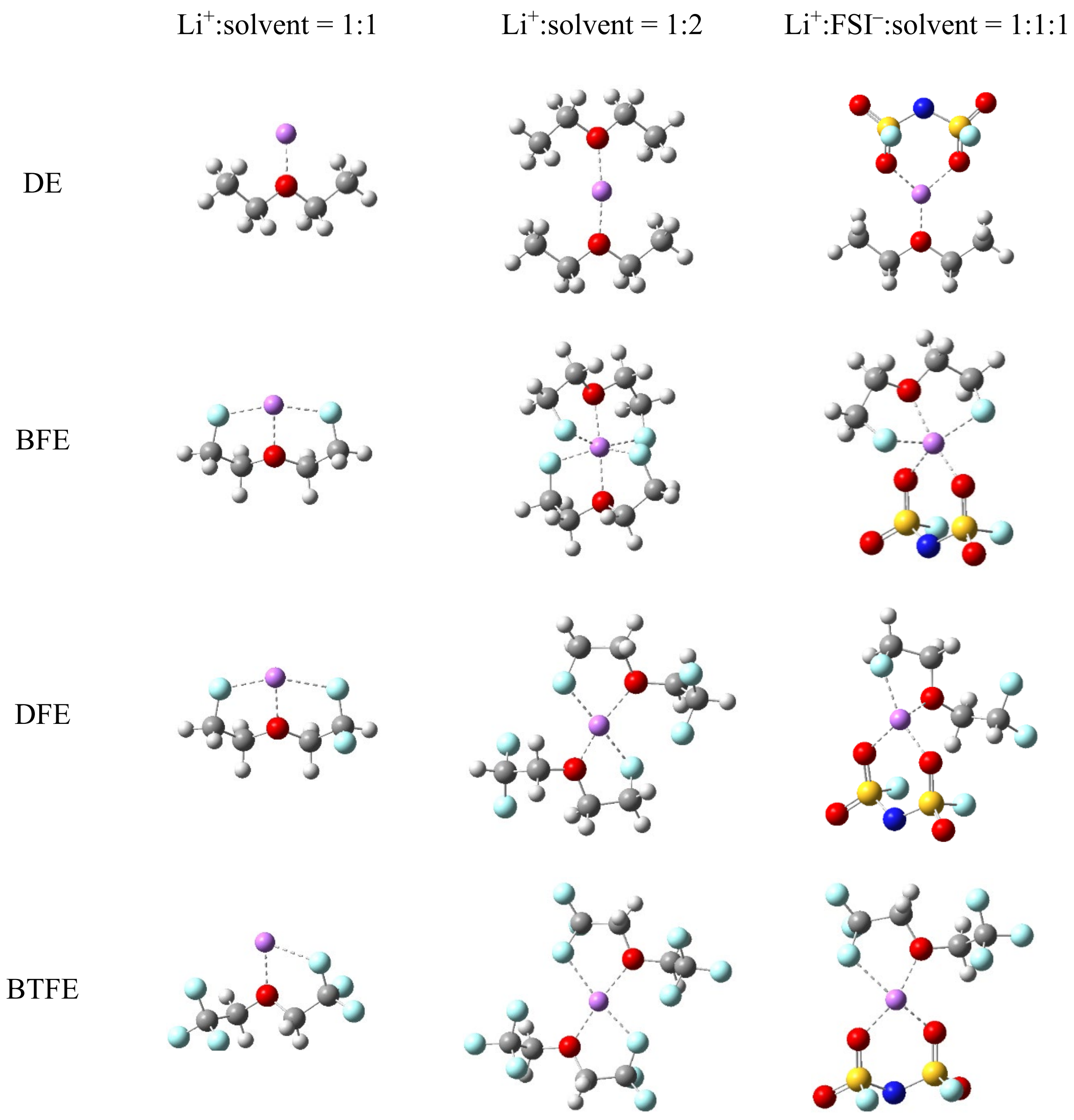

Figure S3. Optimized structures of the Li^+ solvation sheath for the Li^+:solvent = 1:1, Li^+:solvent = 1:2, and Li^+:FSI^-:solvent = 1:1:1 complexes at DEE, FDEE, F3DEE, F4DEE, F5DEE, and F6DEE.

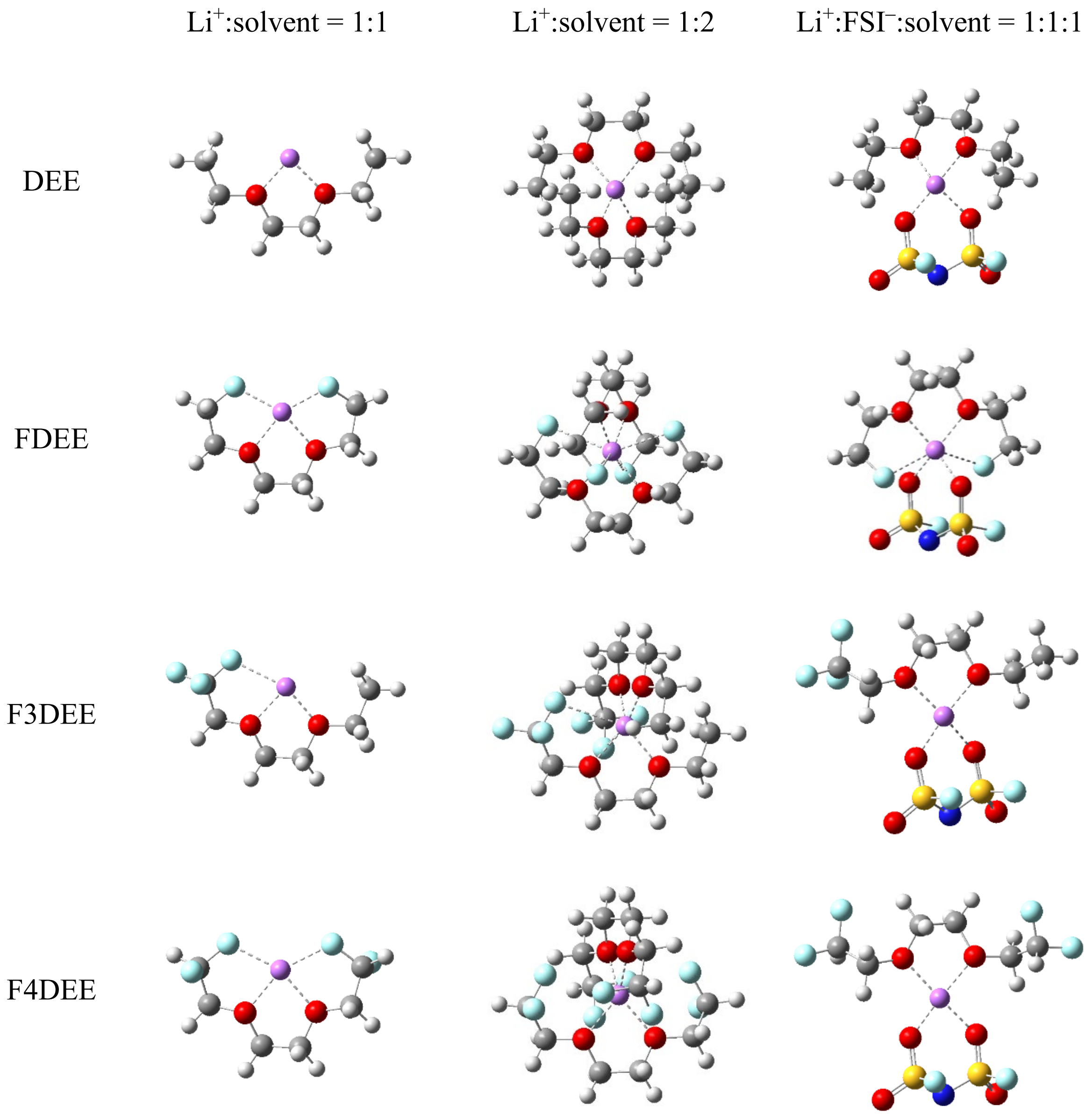

F5DEE

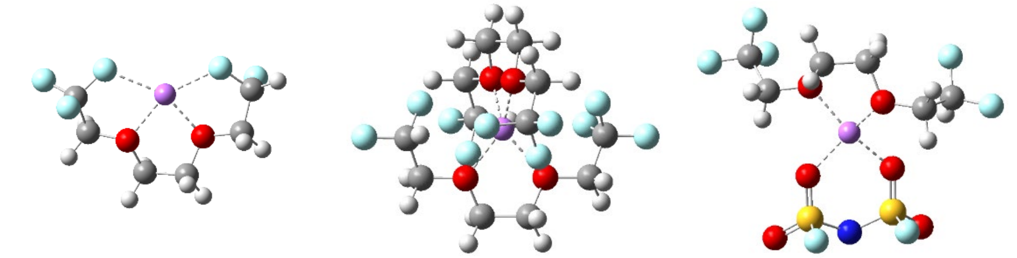

F6DEE

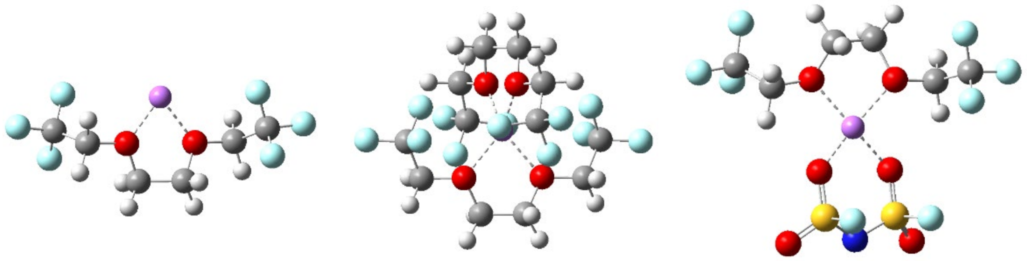

Figure S4. Optimized structures of the Li^+ solvation sheath for the Li^+:solvent = 1:1, Li^+:solvent = 1:2, and Li^+:FSI^-:solvent = 1:1:1 complexes at EA, EFA, EDFA, and ETFA

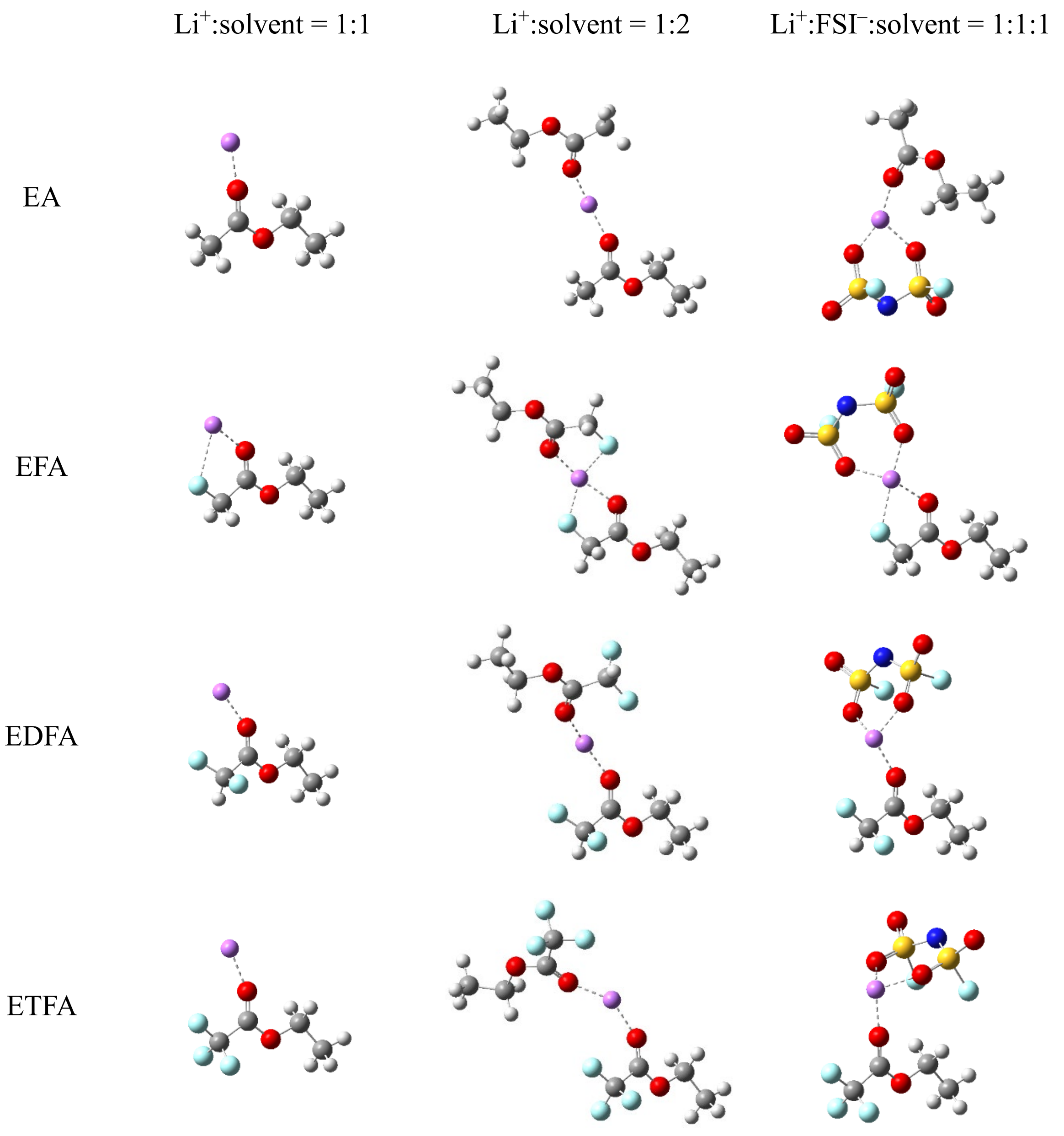

Figure S5. Optimized structures of the Li^+ solvation sheath for the Li^+:solvent = 1:1, Li^+:solvent = 1:2, and Li^+:FSI^-:solvent = 1:1:1 complexes at EMC, F1EMC, F2EMC, and F3EMC.

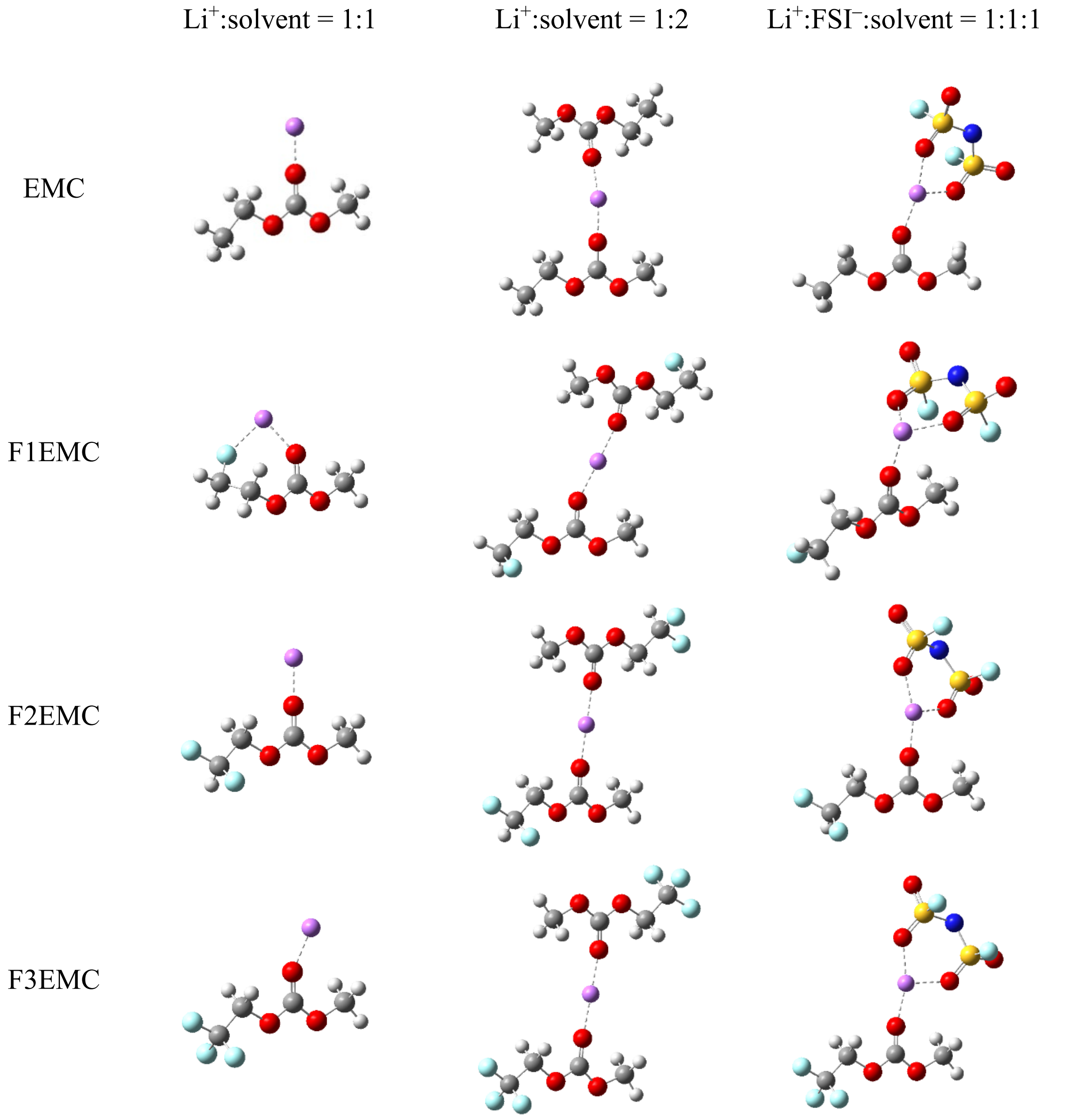

Figure S6. 2D structures and Li^+ solvation energies (Li^+ SE; in kJ/mol) of bis(2-fluoroethyl) ether (BFE) and 1,2-bis(2-fluoroethoxy) ethane (FDEE) for the Li^+:solvent = 1:1 complex.

BFE		FDEE	

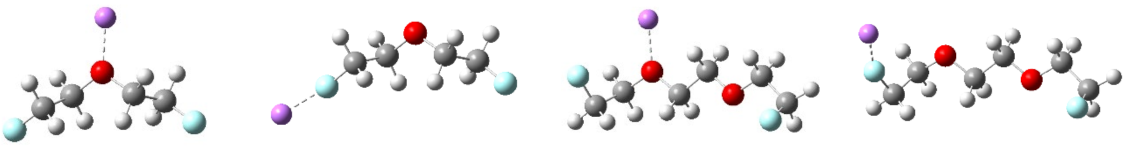

O-coordination	F-coordination	O-coordination	F-coordination
Li^+ SE: 10.6	Li^+ SE: 6.4	Li^+ SE: 33.6	Li^+ SE: 27.6

Figure S7. The NBO charge distribution of bis(2-fluoroethyl) ether (BFE) and 1,2-bis(2-fluoroethoxy) ethane (FDEE) molecules. The numbers indicate the NBO charge of oxygen and fluorine atoms.

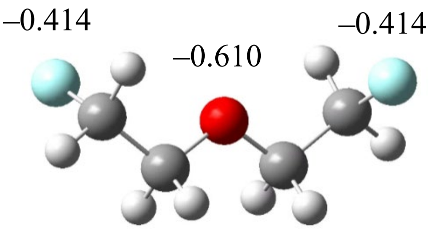

BFE

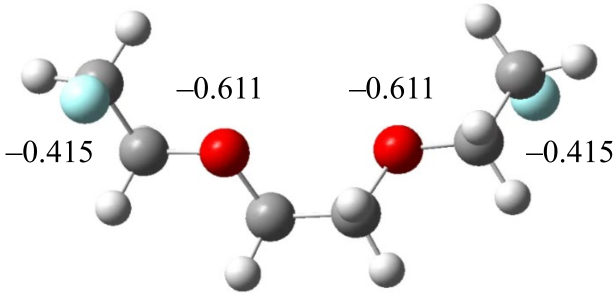

FDEE

Figure S8. 2D structures of the Li^{+} solvation sheath for the Li^{+}:solvent = 1:1, Li^{+}:solvent = 1:2, and Li^{+}:FSI^{-}:solvent = 1:1:1 complexes of bis(2-fluoroethyl) ether (BFE).

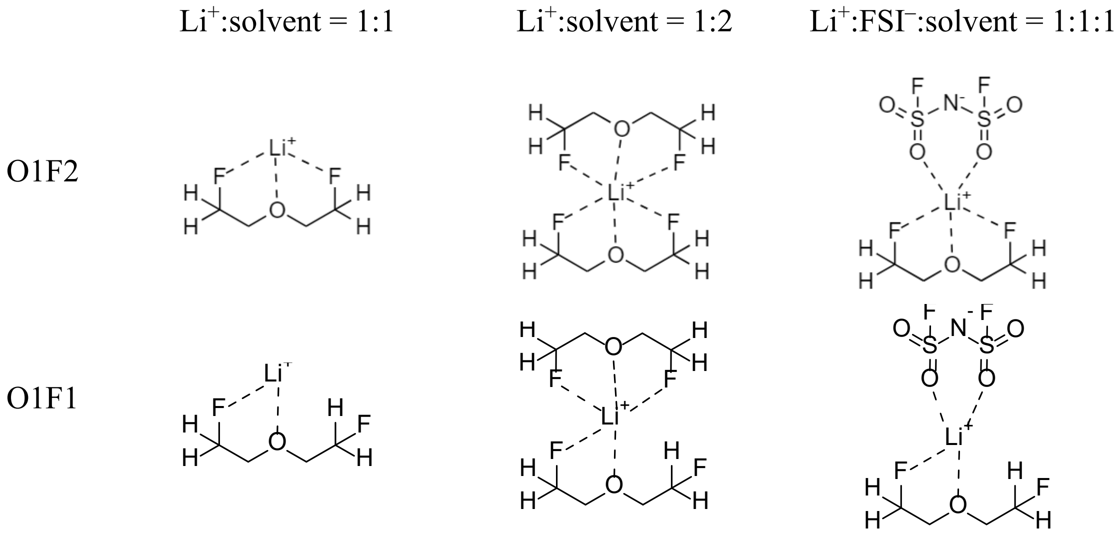

Note: O1F2 refers to a structure where Li^{+} is coordinated to one oxygen and two fluorine atoms, whereas O1F1 refers to a structure where one fluorine atom is excluded from the Li^{+} coordination observed in O1F2.

Figure S9. Li^+ solvation energies of bis(2-fluoroethyl) ether (BFE) for the Li^+:solvent = 1:1, Li^+:solvent = 1:2, and Li^+:FSI^-:solvent = 1:1:1 complexes.

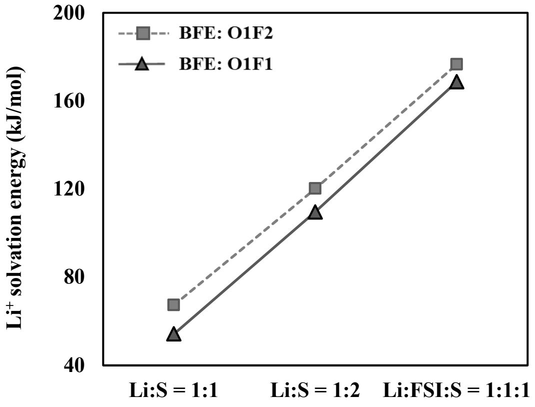

Figure S10. Linear regression plot between the Li^+ solvation energy and ionic conductivity for the Li^+:solvent = 1:1, Li^+:solvent = 1:2, and Li^+:FSI^-:solvent = 1:1:1 complexes at DE, BFE, and DFE.

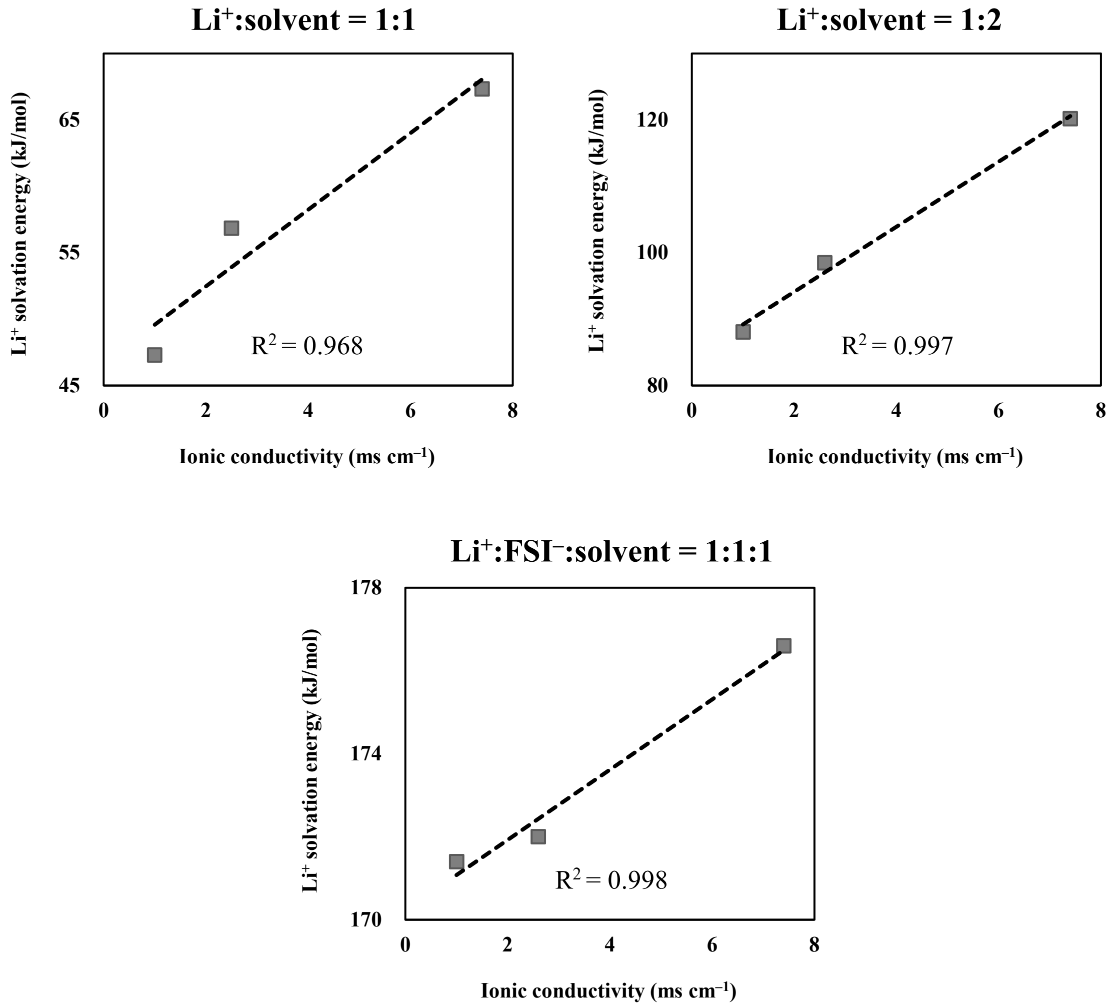

Figure S11. Linear regression plot between the Li^+ solvation energy and ionic conductivity for the Li^+:solvent = 1:1, Li^+:solvent = 1:2, and Li^+:FSI^-:solvent = 1:1:1 complexes at DEE, F3DEE, F4DEE, F5DEE, and F6DEE.

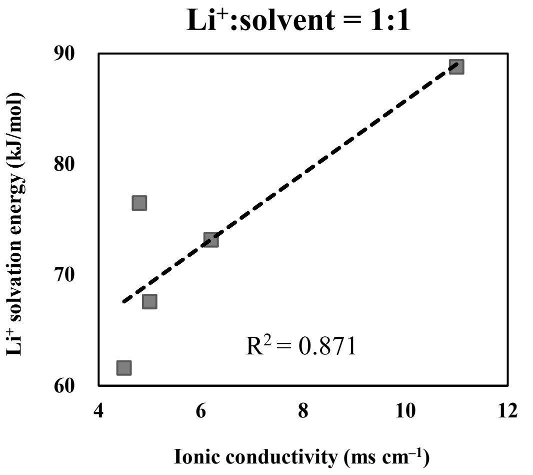

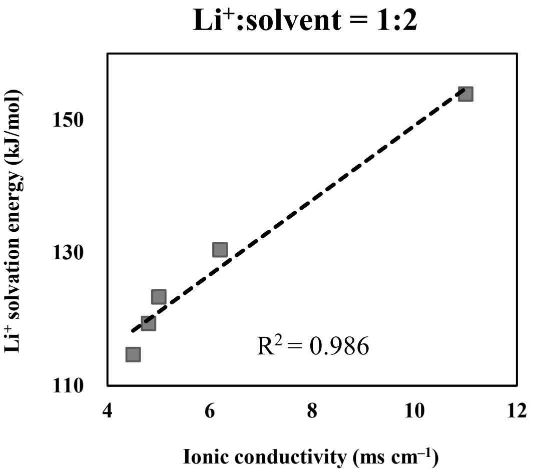

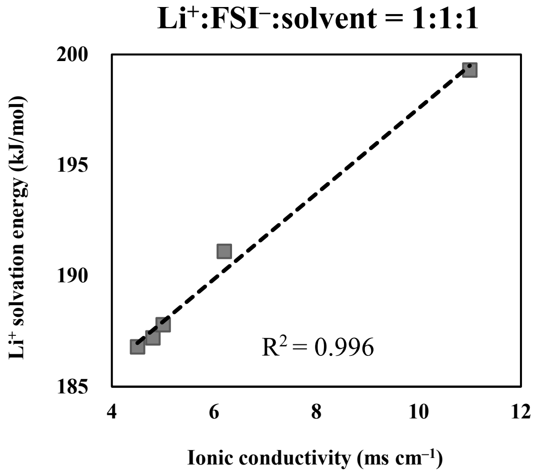

Figure S12. Linear regression plot between the Li^+ solvation energy and ionic conductivity for the Li^+:solvent = 1:1, Li^+:solvent = 1:2, and Li^+:FSI^-:solvent = 1:1:1 complexes at EA, EFA, EDFA, and ETFA.

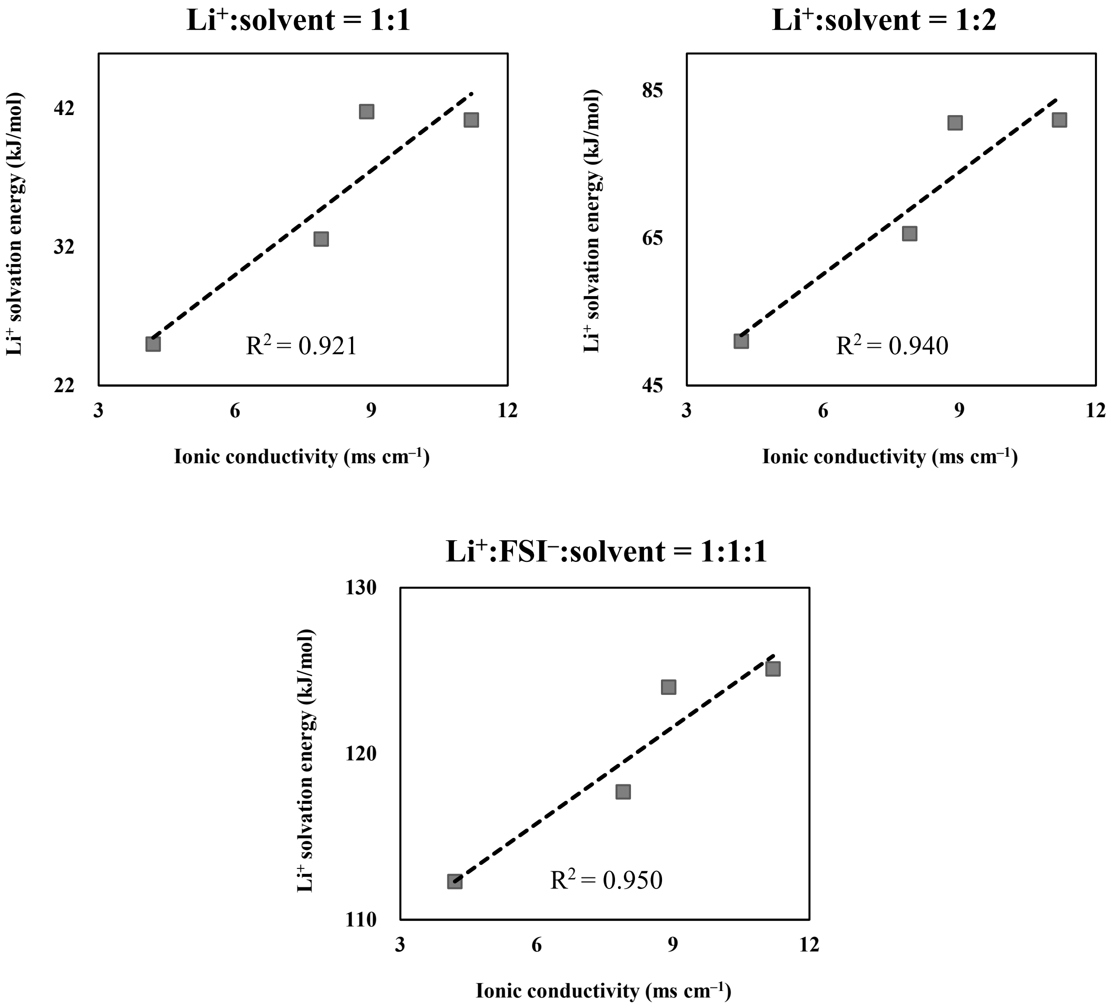

Figure S13. Linear regression plot between the Li^+ solvation energy and ionic conductivity for the Li^+:solvent = 1:1, Li^+:solvent = 1:2, and Li^+:FSI^-:solvent = 1:1:1 complexes at EMC, F1EMC, F2EMC, and F3EMC.

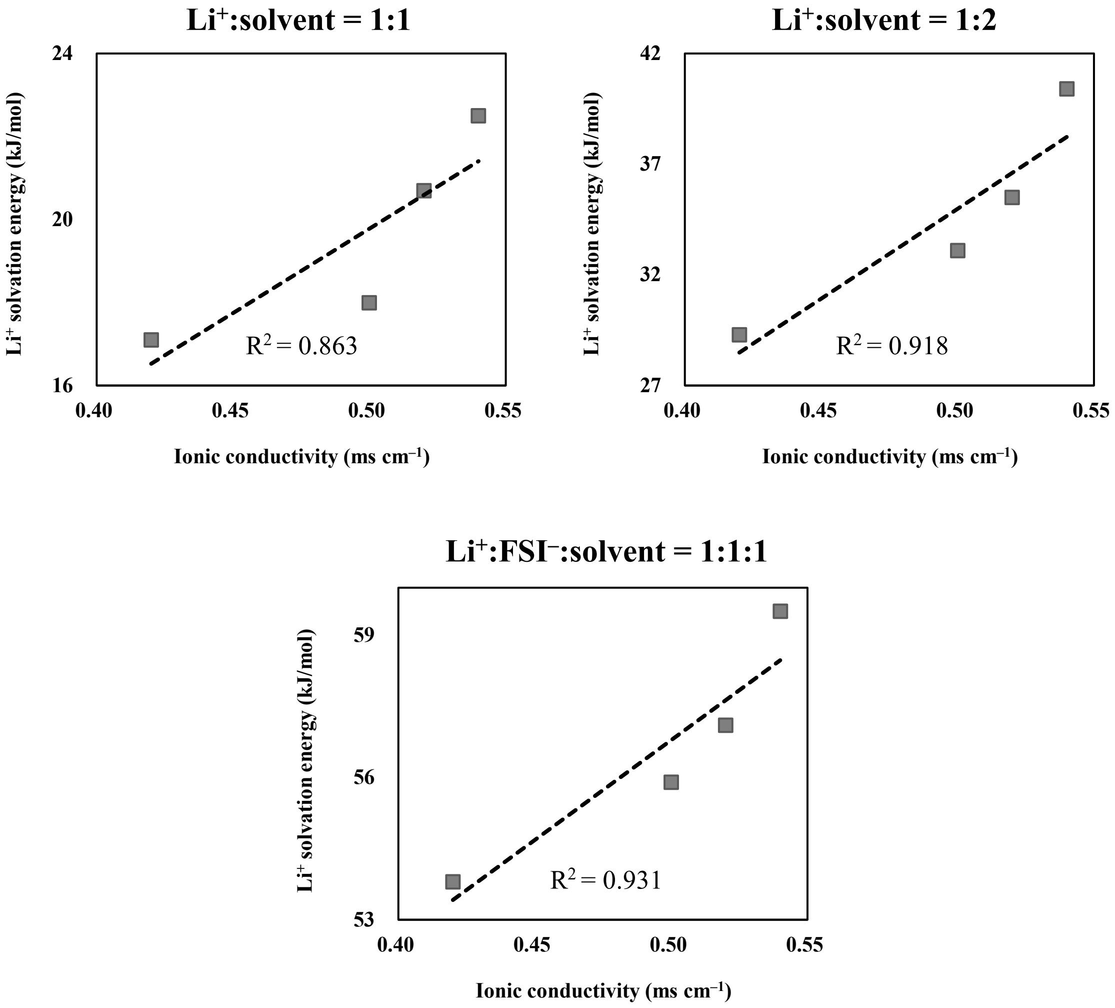

Table S1. The calculated Li^+ solvation energies (kJ/mol) of bis(2-fluoroethyl) ether (BFE) for the Li^+:solvent = 1:1, Li^+:solvent = 1:2, and Li^+:FSI^-:solvent = 1:1:1 complexes. The numbers in parentheses indicate the decrease in Li^+ solvation energy for O1F1 compared to O1F2.

**Molecule**	**Li^+ solvation energy**		
	Li^+:solvent = 1:1	Li^+:solvent = 1:2	Li^+:FSI^-:solvent = 1:1:1
BFE: O1F2	67.4	120.2	176.6
BFE: O1F1	54.2 (–13.2)	109.7 (–10.5)	168.8 (–7.8)

Note: In the structure where one fluorine atom is excluded from the Li^+ coordination, the decrease in Li^+ solvation energy was smaller in the Li^+:solvent = 1:2 and Li^+:FSI^-:solvent = 1:1:1 complexes than in the Li^+:solvent = 1:1 complex. This result indicates that the Li–F interaction is relatively weaker in the Li^+:solvent = 1:2 and Li^+:FSI^-:solvent = 1:1:1 complexes.
